# Legal Aspects of Microalgae in the European Food Sector

**DOI:** 10.3390/foods13010124

**Published:** 2023-12-29

**Authors:** José Diogo Cruz, Vitor Vasconcelos

**Affiliations:** 1 Faculty of Sciences, University of Porto, Rua do Campo Alegre, 4169-007 Porto, Portugal; jcruz@ciimar.up.pt; 2 Interdisciplinary Center of Marine and Environmental Research (CIIMAR), University of Porto, Terminal de Cruzeiros do Porto de Leixões, Avenida General Norton de Matos, S/N, 4450-208 Matosinhos, Portugal

**Keywords:** microalgae, regulation, novel foods, industry

## Abstract

The interest in microalgae as food in Europe is growing due to its remarkable features that can foster a sustainable economy. The lack of tradition on their use among Europeans is changing and a demand for more sustainable products is increasing. The legal framework from the microalgae stakeholders’ point of view has been consistently identified as a bottleneck, regardless of its nutritional value and potential to provide added-value metabolites. Microalgae-based products have been mostly consumed as food supplements, which are characterized by some general uncertainty with regards to food security of products sourced from non-European countries. The novel foods regulation is a landmark in Europe’s food law defining the conditions in which a new type of food can be commercialized. Currently, a more simplified and centralized version is in place, and around eleven microalgae-based products are on the market; however, more than half are represented by *Schizochytrium* sp. derived products (DHA-rich oil). Microalgae have immense potential as a sustainable food source; nonetheless, there is limited experience in assessing the safety of these microorganisms, considering the uncertainty around undesirable substances present in the way they are produced and their diverse metabolites. Here, we overview the regulatory use of microalgae as food in Europe with a focus on market introduction, highlighting the administrative procedures and scientific requirements to assess food safety. We also discuss the implications of the Transparency regulation related to microalgae as novel foods and provide considerations for a more solid interaction between academia and industry.

## 1. Introduction

Microalgae (eukaryotic and prokaryotic photosynthetic organisms) are seen as a key player in the bioeconomy and particularly for the food sector [1]. This sustainable source can be grown in open ponds and closed-well controlled photobioreactors or fermenters to provide nutritious and healthy foods [2]. Germany, Spain, and France represent the biggest microalgae producers, with France representing 65% of *Spirulina* production in Europe [1]. The European Union (EU) food system is highly regulated, and consumer safety is at the base of the General Food Law (GFL) [3]. The safety of a food depends on the associated knowledge gained through technological developments that have led to the implementation of regulatory aspects, with food safety as an intended purpose [4]. Microalgae, often entitled as “the food of the future”, have impressive nutritional value, but there is a lack of tradition with this food for the average EU consumer [5]. The first Novel Foods Regulation (EC) 287/1997 (NFR 1997) set a temporal mark for a mandatory safety assessment prior to the commercialization of food products [6]. Microalgae that have a significant history of consumption proven prior to 1997 within the EU territory were allowed to be marketed without requiring a safety evaluation. This is the case for the species used for commercial products, namely, *Spirulina* (Cyanobacteria), *Chlorella* (Chlorophyta), and *Aphanizomenon flos-aquae* (Cyanobacteria). Microalgae and its metabolites, hereafter microalgae-based products, can be commercialized as the whole cell, ranging from variable nutritional settings to high added value extracts, namely polyunsaturated fatty acids (PUFA), essential amino acids, enzymes, vitamins, carotenoids, and polysaccharides [7]. Microalgae can play a role in upgrading existing food products due to their bioactive and techno-functional properties. Oil/water emulsions, vegetarian food gels, frozen yogurt, dairy products, cookies, biscuits, bread, and pasta have incorporated microalgae-based products influencing protein content, fiber content, and antioxidant properties while emulsifying, foaming, gelation, water, and fat absorption capacities have been noted as techno-functional added properties [8]. Mostly exploited as food supplements, the majority of its production comes from non-EU countries, in particular Asian and South American countries [9]. China itself accounts for more than 90% of the global world production [10]. The introduction of these food products into the EU market is governed by importation laws. Reports on the presence of hazardous substances on commercial microalgae-based products raise concerns on how these laws protect the EU consumer [11].

When microalgae-based products are new to market, the most recent version of Novel Food Regulation 2283/2015 (NFR 2015) applies [12]. A legal framework considers the evaluation of two types of food products: (i) Application of food products that are completely new to the market and (ii) food products that are only new to the EU territory. The former requires an Authorization process which demands exhaustive data on the safety of the food product, while the latter requires a Notification process based on the safety consumption records from a given population for a period not shorter than 25 years. Although the definitions might be simple to describe, they are often complex and laborious to resolve. The European Food Safety Agency (EFSA) works as the safety evaluation entity and provides guidance on these applications despite its case-by-case resolution [13]. High associated costs and time-consuming processes complicate the tracking of small and medium European enterprises, while the microalgae global market is expected to reach USD 4.7 billion by 2027 with an estimated CARG (Compound Annual Growth Rate) of 4.3% [14]. Moreover, the introduction of Regulation (EC) 2019/1381 (Transparency Regulation) has come to amend the GFL and applies to all sectoral rules including novel foods. It intends to bring clarity among EU consumers by demanding a more transparent management of risk in the food system chain [15]. 

Industry and academia have common playgrounds; however, closer relationships are needed next to legislators. The EU has been putting into practice strategies and plans to stimulate what are seen as key players in the bioeconomy [16]. The INTERREG Atlantic Area EnhanceMicroAlgae (EMA) project EAPA_338/2016 (2017–2020), further extended until June 2023, contributed to the competitiveness of the microalgae-based industry in the Atlantic Area through the transfer of technological and economic expertise to the commercial sector [17,18]. Furthermore, it promoted the creation of an online stakeholder database, a decision support tool to help SMEs on the culture system, training sessions and workshops, a series of comics and illustrations on microalgae, and finally the deployment of a marketplace as a platform for the exchange of services and interests [19]. In this context, we aim to enhance the outcomes of the project by establishing a gateway to legal information related to the microalgae food industry and by presenting thoughtful considerations on this emerging topic. 

Note 1: Throughout the document, we refer to microalgae-based products as a group of photosynthetic eukaryotes and prokaryotes (Cyanobacteria); the protozoan class Labyrinthulomycetes [20,21] is also included in this large group, since it is also considered in the scope of the technical group European Committee for Standardization Technical Committee (CEN TC)/454 “Algae and algae products”, which aims to deliver European standards for the algae industry [22]. 

Note 2: The present work does not deal with animal feed, despite the overlap of the topics, nor with health claims or labelling.

## 2. Food Law Foundations in the EU

The need to guarantee food as safe for EU citizens led to the creation of Regulation (EC) 178/2002, also known as the GFL, wherein the general principles, requirements, and procedures for food and feed at the Union level are laid down. This law is the cornerstone of Europe’s food system, and its extension does not only apply to the food product itself but also to any component that interacts with the food supply, such as contact materials, solvents, adjuvants, etc. The same regulation gave rise to the EFSA, which is responsible for risks associated with the food chain [3]. The EFSA provides advisory actions and delivers scientific opinions which are then used for the generation of European policies and legislation [23]. In addition, the management of emergencies and crises is safeguarded by the generation of main procedures for the Rapid Alert System for Food and Feed (RASF). This tool can be used by any food business operator (FBO) and aims to prevent harmful food risks to European consumers [3]. In 2019, a RASF alert related high levels of sulphites in *Chlorella* biomass was reported; however, it was proved to be naturally occurring (Directorate-General for Health and Food Safety) [24]. The GFL is the ground base for several sectoral legislations, defining when a product is new to the market and in which conditions it is considered safe, as is the case for novel foods [25].

## 3. Novel Foods

A novel food is any product with no significant degree of consumption before 15 May of 1997 and which was completely new to the European population [6]. NFR 1997 was valid until 31 December 2017 and was elaborated to protect consumers and harmonize rules between Member States (MS). 

The NFR 1997 version was an applicant-based system, in which Authorization was only granted to the applicant. Subsequent applicants could undergo a shorter bureaucratic process based on food equivalency. The divergent interpretation of MS to novel food definitions and issues associated to the safety of genetically modified food forced the actualization of this regulation [25]. On 1 January 2018, regulation NFR 2015 took effect, which also allowed recent scientific and technological breakthroughs to be considered. Art. 3(2) of NFR 2015 presents expanded categories of foods, including those originating from plants, animals, microorganisms, cell cultures, and minerals (e.g., algae, insects, vitamins, minerals, food supplements, etc.). In addition, novel foods may also mean any food resulting from a non-conventional production process and food consisting of engineered nanomaterials. Moreover, a product-oriented application was established by the EFSA including a simplified process called Notification for food only new to the EU territory, also known as traditional foods from third countries [12]. These two application processes are currently in place (Authorization and Notification) and can be electronically submitted to the European Commission (EC) services [26]. Furthermore, institutional (EU countries, non-EU countries, and the Commission) and non-institutional (FBO) applicants are entitled to apply, and Art. 3(2)(d) NFR 2015 foresees that national competent authorities may aid different interested parties [12]. For instance, for those that are active in the production of the same species, the preparation and submission of an application can take place together. In this way, joint applications are allowed if the product of interest shares similar characteristics; it is up to the applicants to clarify their cost–benefit sharing, confidentiality, regulatory data protection, and antitrust compliance. The EC is empowered with the initiative to update novel food Authorization matters whenever needed. In the same way, the EC is assisted by the Standing Committee on Plants, Animals, Food and Feed (SCoPAFF), which is composed of all EU countries and led by an EC representative. The SCoPAFF delivers opinions to the EC to promote a better legislative implementation [27].

The need to properly legislate novel foods has excluded food enzymes (Reg. (EC) No. 1332/2008), food additives (Reg. (EC) No. 1333/2008), food flavourings (Reg. (EC) No. 1334/2008), and GMO food and feed (Reg. (EC) No. 1829/2003) due to the complexity of each category. In addition, the fact that a novel food is authorized to be placed on the market does not imply that sectoral rules cannot apply, as is the case for food supplements (Directive 2002/46/EC), fortified food (Regulation EC No. 1925/2006), and special foods (Regulation EU No. 609/2013).

### 3.1. Microalgae-Based Products on the Market before 1997

As a starting point, the EC provides a non-exhaustive list of products, including microalgae-based products, that are sensible to the NFR known as the Novel Foods Catalogue (NFC) [28]. Compiled based on MS feedback, it identifies the current status of the product (novel, not novel, not novel to food supplements, or ongoing enquiry process) and if the case falls under the scope of the NFR. However, if a microalgae-based product is not on the NFC, it does not mean it is novel; instead, it can mean that its status is not yet addressed, or that an enquiry was not requested under Art. 4 of NFR 2015 [29]. Microalgae-based products that were significantly consumed within the EU before 1997 are not subject to NFR 2015 (see Table 1). As a matter of fact, the resources available at the time for identifying which microalgae-based products had a significant degree of consumption in the EU were limited due to their limited usage. It is possible that more microalgae-based products than the ones listed in Table 1 were on the market in certain MS before 1997. As an example, both *Aphanizomenon flosaquae* (AFA) var. *flosaquae* and *Dunaliella* (β-carotene extract source) cyanobacteria and microalgae, respectively, were not grown in any European territory, yet they were commercialized at a large scale worldwide as food supplements long before 1997 [30,31]. The fact that AFA is included on the NFC as not novel and *Dunaliella* is not shows that it might not have been addressed or requested, even though their commercialization in health food European shops was noted. 

The JRC technical report on algae as food and food supplements in Europe provided a comparative list of microalgae-based products within the NFC, the Union list of authorized novel foods and official MS for food and food supplements, aiming to reduce ambiguity among species allowed. This work highlighted repetitive entries and limitations on the taxonomic designations of certain microalgae, and an update on the NFC for the categories of food and/or food supplements is proposed [29]. As an example, the commercial name of “Spirulina” overlaps with genus of *Spirulina* and *Arthrospira*. Although *Arthrospira platensis* (included on NFC) is regarded as frequent food item, recent revisions have found evidence of a history of consumption in Europe of *Limnospira maxima* and *Limnospira fusiformis*. On one hand, official lists of France, Italy, and Belgium include *Limnospira maxima*, while on the other hand *Limnospira indica* and *Spirulina major* are only included in French records [29]. Thus, this report is of extreme relevance to the algae sector, and indicates that taxonomic updates and unspecified species of certain genus are constraining the use of microalgae as food and food supplements. In addition, it is a fact that harmonization between MS is difficult, and thus it is always recommended to consult national competent authorities prior to commercialization, when possible, with the aid of taxonomists and standards [32] for species identification. Overall, advancements in DNA sequencing, bioinformatic tools, and taxonomy approaches explain the evolution of names of microorganism, as is the case of *Chlorella* sp. [33]. These changes aim to reflect a more accurate and up-to-date representation of their evolutionary relationships and characteristics, yet they can be seen as a limitation for market application.

When there is absence of evidence of safe consumption and use as food supplements, such as the case of widely used research model *Chlamydomonas reinhardtii*, the novel food enquiry requires additional safety evaluation [34]. Moreover, products derived from foods that do not fall within the scope of novel products, generally known as high added value products, are not excluded from NF evaluation. This is the case for phycocyanin, a blue pigment from Spirulina [35]. Blue Spirulina Powder is a product from Rawnice, composed of phycocyanin. Based upon information from the Food Administration of Sweden, Blue Spirulina Powder cannot be commercialized within the EU as food, yet it is allowed for outside EU (e.g., North America, Asia, and Australia) [36]. Simultaneously, the commercialization of algal oils derived from species present in Table 1 will need to follow an NF evaluation. 

#### 3.1.1. Food Supplements

Food Supplements were first regulated by Directive 2002/47/EC. The directive only lists recommended composition of sources for vitamins and minerals. Other substances, such as amino acids, plants, extracts, and other biologically active substances, as well as their purity are controlled independently by each MS. The MS approaches can vary from positive/negative lists to Authorization procedures on a case-by-case basis. In addition, since there is no regulation on these substances, no limits of use are established [37]. Harmonized regulations can promote a better EU environment aligning MS to the concerns of the commercialization of food supplements. When a food supplement is new and posterior to 1997, its introduction as a food supplement falls within NFR 2015 and a different scrutiny is applied [12]. A recent survey on algae commercialization as food supplements has shown an impressive number of 27 microalgae-based products (before 1997) wherein some genera have no mention in the NFC [5,29]. Almost 50% of these products correspond to revised genus and species of commercial names of *Chlorella* and *Spirulina*. Other genera, herein referred to by their commercial names, are or can be commercialized in the form of extracts, making them high-value products, such as *Haematococcus* (astaxanthin, lutein, omega-3, omega-6), *Dunaliella* (α and β-carotene, lutein and omega-3), *Nannochloropsis* (omega-3), *Chlorella* (protein, glucans, omega-3), and *Spirulina* (protein, β-carotene, omega-3, omega-6) [38]. Some are post-novel food market approval while others, such as the Scenedesmus genus, are documented in markets of countries such as Russia, Germany, Austria, and Switzerland [5]. 

The importation of microalgae-based products for food from non-EU countries has raised safety issues. These fall within the category of food of non-animal origin (FNOA) and therefore require a phytosanitary certificate. Briefly, this certificate guarantees the traceability of the exporter until its destination. Additional requirements might be needed in cases where the product is associated with an emerging risk due to its place of origin and/or susceptibility to pests [39]. However, in EU law, there is no reference to microalgae-based products related to this type of risk [40]. 

The quality of these supplements has been often questionable among the scientific community [41,42,43,44,45,46]. Several reports have called attention to potential hazardous components in Cyanobacteria- and/or *Chlorella*-containing food supplements sold in the European market coming from non-EU countries [11,47,48,49]. Cyanotoxins, such as Microcystins, are potent hepatotoxins produced by several cyanobacteria (*Planktothrix*, *Microcystis*, *Aphanizomenon*, *Nostoc*, and *Anabaena* genera) [50]. The production of this type of toxins is exclusive to cyanobacteria; however, *Chlorella*-based products have been identified with microcystins [11,37,51]. These relatively stable cyclic heptapeptides resistant to environmental factors which act as inhibitors of protein phosphatases 1 and protein phosphatases 2A for mammals and higher plants. Their detection methods are well studied; however, the number of variants (270 known) makes their analysis more efficient when mass spectrometry is coupled with traditional chromatographic methods (LC, GC) while HPLC techniques imply the use of standards which are not always available [52]. Biological contamination with other organisms such as fungi hyphae, fungi conidia, or other algae and bacteria can also be found, wherein non-desirable organisms are more frequent in natural production facilities such as lakes and open ponds [11]. Moreover, heavy metals, pesticides, mycotoxins, antibiotics, and polycyclic aromatic hydrocarbons can also be present due to anthropogenic causes [47]. Microalgae can bioaccumulate heavy metals, raising concern about the toxicity of microalgae food supplements. In fact, the accumulation of heavy metals in microalgae can integrate in either organic or inorganic form with different toxicity levels, with the organic form less prone to being toxic to humans [49]. This indicates that maximum levels of the presence of different forms of metals in microalgae-based products should be further studied. 

In addition, the lack of transparency of products labelling with composition and production conditions has been confirmed, but not consistently [11]. Nonetheless, nutritional and safety variations between batches have been pointed to as a reason for process optimization in a world-wide *Chlorella*- and *Spirulina*-based products assessment [47].

## 4. Novel Microalgae-Based Products

Bringing a new microalgae-based product to the market requires compliance with the novel food scope and attribution to at least to one of the 10 categories by means of a public consultation [12]. Based on the principles of the GFL, the commercialization of a novel food shall (i) not pose risk to human health, (ii) not be misleading to the consumer (e.g., replacing a food with a change in nutritional value), and (iii) not prove to be nutritionally disadvantageous when it intends to replace an existing food [3]. 

The Commission Implementing Regulation (EU) 2017/2470 provides a list of all authorized novel foods that can be commercialized within the EU [53]. Entitled the Union list, it corresponds to a dynamic repository of all approved novel foods and their associated conditions of use, additional labelling requirements, and post-market monitoring requirements [54].

One of the latest amendments for microalgae-based products specifies conditions for the placing on the market of dried *Euglena gracilis* [55]. A list of microalgae-based products authorized as novel foods, including whole foods and high value extracts, is included in Table 2. Data protection on *Euglena gracilis* provides exclusive product commercialization in the EU market by a non-EU enterprise. 

The following three subsections disclose administrative procedures from the novelty enquiry and the Authorization and Notification procedures.

### 4.1. Consultation Process 

If the FBO is not sure whether its microalgae-based product is novel, an inquiry process should be submitted to a competent authority of an EU MS where commercialization is intended.

The placing on the market of novel foods is defined according to Art. 4 (1) of NFR 2015, and a recommended template for the consultation process is provided by the Commission Implementing Regulation (EU) 2018/456 [57]. The evaluation documents include a cover letter, technical dossier, supporting documents, and an explanatory note clarifying the purpose and relevance of the submitted documentation. Briefly, once the consultation is validated, the MS has the duty to inform all interested parties (the FBO, Commission, and other MS) that a four month evaluation will proceed. After that period, the evaluation can be extended for an extra four-month period, and in the meantime the MS can request more information from the FBO to help the decision of the Commission and other MS. The final decision is shared among the interested parties and the Commission publicly announces the Novel Food Status [12]. 

An example list of the national competent authorities of Atlantic Arc authorities (countries involved in the EnhanceMicroAlgae project) is presented in Table 3. An exhaustive list of national competent authorities is provided by the EC website [58].

Note that this decision is done on a case-by-case basis. For instance, if a similar product is already on the market, the final product composition, species used, and production process must align with the one already on the market. Otherwise, this product might be easily considered as novel. Another particularity of this consultation process is that FBO must choose one country for the enquiry process, even if its intention is to commercialize in a range of countries. 

Confidentiality, Art. 26 of NFR 2015, can be requested as mentioned before; however, the following information is required to provide a publicly available resume of the application: (a) name and address of the applicant; (b) name and food description; (c) a resume of the studies presented by the applicant; (d) case analysis methods. The consultation process can be cancelled by the applicant for a period of three weeks, a period in which the confidentiality is active. Given the novel foods status attributed, the FBO should be aware of both processes of permission to commercialize, namely Authorization and Notification [59,60].

### 4.2. Authorization Process

Art. 10 of NFR 2015 defines the Authorization process as the submission of the dossier to the Commission, which includes the documents listed below. Bold entries correspond to the information used for summarizing the request to the MS and be shared to the general public.

- **Name and address of applicant;**
- **Name and description of novel food;**
- **Description of the production process;**
- Detailed composition of the food, specifications, batch-to-batch variability, stability;
- **Scientific evidence that novel food is not a safety risk for human consumers;**
- **Anticipated intake level;**
- Analysis methodology;
- Intended use and labelling requirements.

The EC is responsible for the validity check, which involves the verification of compliance with the abovementioned data. This step can involve the aid of the EFSA for a period of 30 days maximum, referred to as a suitability check. Once past this phase, concerns on human health effect are approached through a risk assessment carried out by the EFSA, which has nine months to deliver its opinion. For cases in which the information provided is not sufficient to prove the safety of the novel food, the EFSA can agree with the FBO for an additional period. It is important to stress that the FBO can quit the application at any time. If the novel food is considered safe for human consumption, the Commission has seven months after the EFSA opinion to present an execution act to the SCoPAFF committee in which the novel food can be commercialized within the Union, and the Union list is updated. Once approached, the Commission can officially confirm the legality of this food product on the market. For cases in which the EFSA’s opinion was not requested, the seven months start counting when the Commission receives the application [59]. The Commission Implementing Regulation (EU) 2017/2469 provides the templates to guide the FBO in delivering the scientific and administrative requirements for Authorization [61].

### 4.3. Notification Process

Traditional foods from third countries are considered foods that were part of a country diet of a significant number of people for a period not shorter than 25 years [12]. A traditional food must be derived from primary production, and microalgae cultivation and wild harvesting are included. According to Art. 14 NFR 2015 the FBO shall provide the following documents via e-submission:

- Name and description of the FBO;
- Name and description of the traditional food;
- Detailed composition of traditional food;
- Origin country;
- Documented data relative to the safe history of consumption in the third country;
- Proposal of the conditions of use and the required specification for the proper labelling of the traditional food.

**Figure 1:**
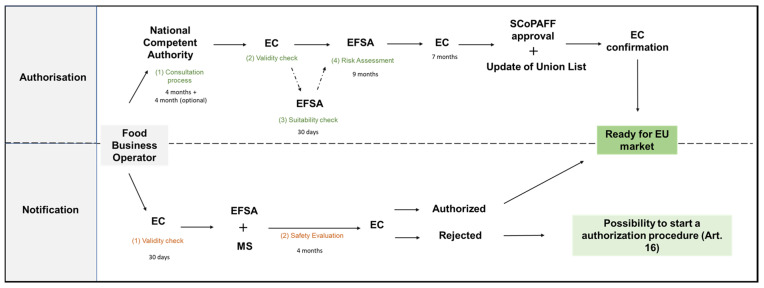
Schematic representation of the different stages involved in the Authorization and Notification procedures under NFR 2015.

The Commission Implementing Regulation (EU) 2017/2468 provides templates to guide the FBO in delivering the scientific and administrative requirements for the Notification [62]. The Notification is addressed to the Commission which, after a validity check of no longer than thirty days, forwards it to the MS and the EFSA. An examination period of four months is dedicated to the MS and the EFSA concerning the safety of the food. Based on the outcome, the Commission informs all the parties regarding the approval of the traditional food. If the decision is favourable, the traditional food can be marketed in the EU, and the Union list is updated. For cases where the Commission does not approve its commercialization due to safety matters, the FBO can appeal for an Authorization process in accordance with Art. 16 NFR 2015. At this point, the FBO can complement the provided documents with data addressing the objections raised forwarded back to the EFSA [60]. A brief review of the Authorization and Notification process under NFR 2015 is shown in Figure 1.

## 5. EFSA Evaluation Main Guidelines

NFR 2015 brought advantages to speed up the application process, among them adding the EFSA as the authority responsible for evaluating the safety of novel or traditional foods [12]. Prior to the introduction of NFR, i.e., at the time of NFR 1997, the risk assessment was performed by each MS, while the EFSA would only conduct a complementary assessment upon EC request. Currently, risk assessment is performed by the EFSA, in particular by the Panel on Nutrition, Novel Foods and Food Allergens (NDA) [53,63]. This panel comprises a group of experts responsible for evaluating the information provided by the FBO and determining if the proposed food is safe to be in the EU market [64].

The compilation of a dossier for both Authorization and Notification are predominantly based on the literature review, meaning that the sources and the methods applied to obtain defined information are of uttermost importance and should follow systematic review principles. In the same way, the EFSA sets out how and where the analysis/tests should be performed by accredited laboratories, sustained by official guidelines (e.g., OECD, EMA, and ICH) and quality systems (e.g., GLP, GMP, GCP, and standards such as ISO, CEN). Moreover, the EFSA supports Directive 2010/63/EU on the protection of animals used for research and development purposes, and recommends to replace, reduce, or refine and avoid duplications whenever possible. The applicant can provide non-requested information; however, its utilization should be properly justified. Protected data can be validated by the EC under specific conditions defined by Art. 26 of NFR 2015, including data considered decisive for the risk assessment. The EFSA risk analysis will determine if it is possible to assess the safety of the novel food without the data and provide an opinion on its relevance as protected data [65].

The dossier follows an objective structure where administrative data of the applicant and characterization of the novel food are detailed. The administrative section includes common information for both applications, like an index, company name, contact person, and the regulatory status of the novel food outside the EU. The technical characterization should provide all the data proving the safety of the food [66]. 

The two forms of application have different compilation requirements, with the Authorization being the most extensive dossier (see Table 4). Both applications contain common information related to the novel food source, basics of the production process, and main compositional features. Microalgae-based products must clearly identify the scientific name of the producing organism, potentially to the species level. ALGAEBASE is an important open source of taxonomic information based on reference strains and is accepted as an official algae database [67]. Culture collections are fundamental to preserve the biodiversity and work as biobanks of economically important species [68]. The legality of the organism, considering other international agreements such as the Nagoya Protocol, strengthens the origin of the novel food [69].

Microalgae-based products can be obtained from different modes of cultivation (i.e., photo-autotrophic, heterotrophic, and mixotrophic mode), while the production processes can range from uncontrolled open systems to highly controlled closed systems such as fermenters. All the inputs must be considered, even the ones that are not necessarily intentionally added, as is the case for open systems exposed to substances from the surroundings of the production facility. Water and nutrients source are comprehensively evaluated and compared to byproducts, impurities, and residues from seeding to final product packing. The international quality standards mentioned before are a seal of confidence for the extent of analysis required. 

Whole cell product implies a different obligatory scientific proof in comparison to extract level, due to the increased number of metabolites. Compositional data relate to a qualitative and quantitative analysis in terms of physico-chemical and biochemical properties, as well as microbiological characterization. In addition, final product stability studies under abiotic and biotic factors must be presented in the same way. The specification of the novel food aims to set a quantitative limit whenever nutritional and/or biologically active components are present. 

The history of use of the novel food and/or its source in the Authorization dossier is analogous to the data from experience of continued use in the Notification process. Closely related foods products or sources play an important role in data collection for both literature reviews. For Authorization of microalgae-based products, the presence of critical substances, potential hazards, and precautions are mapped, while available data on the food product usage outside the EU and/or its non-food application are relevant for the risk assessment. Under the Notification process, a detailed characterisation of the population, consumption habits, and associated risks (allergenicity, toxicity, etc.) are required, and can be obtained from a wide range of relevant sources from scientific literature to cooking books [70].

The proposed uses, use level, and anticipated intake comprise the last category in the Notification process. Estimation of intakes to a specific population are characterised by form of use, food categories, intention to substitute for an already existing food, maximum amounts, and average daily intakes [71]. The interaction with other substances (e.g., food supplements) is considered in the same way, as the cumulative effect of undesirable substances may impact proposed intake levels. Absorption, distribution, metabolism, and excretion (ADME), nutritional information, and toxicological information complement comprise a detailed and expensive set of analysis for the conclusion of a safety assessment for the Authorization dossier. Part of a report requested in 2017 by the EC Directorate General for Energy provides good practices on the development of a strong dossier which should not be disregarded. An estimation of the cost associated with most important toxicity assays is set to vary between EUR 50,000 to 200,000 depending on the service provider and the number of batches [53,63].

### Qualified Presumption of Safety (QPS)

Foods isolated from microorganisms, as is the case for microalgae-based products, demand clear organism identification and association with a safe status. Similar to the USA’s Generally Recognized As Safe (GRAS), the EFSA introduced a concept in 2007 known as QPS, which is a slightly different concept since it confers the decision role to the risk assessors instead of to the FBO. In addition, the absence of acquired antibiotic resistance and virulence factors are criteria highly considered in the attribution of the QPS status, unlike the GRAS [72]. The QPS is defined as “an assumption based on reasonable evidence while the qualification stands as a remark for exceptions to happen” [73]. The recommendation of an organism for a QPS taxonomic unit means that the organism has an unambiguous identification to the species level and its main characteristics are outlined. The absence of a clear taxonomic identification exempts the organism from being considered for the QPS list. The risk assessment is completed using a body of knowledge that includes history of use, scientific literature, clinical aspects, industrial applications, ecology, and the safety of the end use product [53,63]. From 2017 to 2019, species such as *Aurantiochytrium limacinum*, *Euglena gracilis*, and *Tetraselmis chuii* were recommended in the QPS list “for production purposes only” [74]. The red algae *Galderia sulpharia* was recently evaluated; however, the lack of a consistent body knowledge excluded this species from a QPS status [75]. Microalgae species closely related to the abovementioned QPS taxonomic units are good candidates for potential inclusion on the QPS list. More recently, QPS status for closely related species was evaluated, namely, *Aurantiochytrium mangrovei*, *Schizochytrium aggregatumis*, *Chlamydomonas reinhardtii*, and *Haematococcus lacustris* (former *Haematococcus pluvialis*). Due to the available data, QPS status was only provided for *Haematococcus lacustris* (for production purposes only, meaning no viable cells of the production organisms can be present in the final product) [76].

## 6. Transparency Regulation Implications to the NFR

The EU has set a new regulation on the transparency and sustainability of the food risk assessment chain, which entered into force on 27 March 2021. The Transparency Regulation is a fitness check for the GFL that applies to several sectoral rules, in particular NFR 2015. Briefly, this regulation intends to clarify and provide trust to EU citizens while scrutinizing industry breakthroughs, not forgetting the importance of confidentiality. The Transparency Regulation introduces sectoral changes, such pre-advisory meetings to applicants, Notification studies, third party consultations, verification studies, and disclosures of potentially sensible information. It amends NFR 2015 in particular related to the administrative parts of Art. 10, 15, 16, and 23. In detail, one of the key resolutions is the centralisation of the EFSA importance through the creation of a pre-submission phase in which all the studies commissioned/carried by a future applicant or EU laboratory shall be notified to the EFSA which will be used to feed a publicly available European Database. It is important to stress that only after the submission has been accepted or officialised are the data made public. Moreover, this step also includes optional advisory pre-submission meetings with EFSA staff, aiming to help future applicants, in particular from small and medium enterprises (SMEs); however, advisors shall not coincide with the evaluation panel. Whenever the FBO has sensible information on the application, it shall deliver two versions to the EC: a confidential version and a non-confidential version. Based on pre-defined criteria, the EFSA will have 10 days to decide on the confidentiality of the provided information. Whether the request falls under the confidentiality criteria, the FBO has two weeks to deal with the EFSA decision and opt for the most convenient resolution. The process continues through a public consultation to scrutinise other relevant data of which the EFSA might not be aware. Extensions are also granted on this regulation in which a pre-notification of the studies planned shall be shared with the EFSA in advance and made public to third party scrutiny. As mentioned, confidentiality is determined by the EFSA on a case-by-case basis; however, pre-defined criteria protect manufacturing processes, industrial and technical specifications, in-house analysis methods, personal data, protocol for efficiency studies, intellectual property, exclusivity data, and general data protection. It is noteworthy that the Transparency Regulation can unlock confidential information conclusions on the safety of a product when potential impacts to human and animal health, as well as to the environment, are present [77,78,79].

Administrative procedures for the Authorization and Notification of novel food Commission Implementing Regulation (EU) 2017/2469 (see Section 4.2) were updated by the Commission Implementing Regulation (EU) 2020/1772, while for traditional foods the Commission Implementing Regulation (EU) 2017/2468 (see Section 4.3) was updated by Commission Implementing Regulation (EU) 2020/1824. In short, both implementation acts were amended with the Transparency Regulation, implying normalization on the presentation of requests, and that EFSA’s notified studies, in depth justification of confidential data, and outcomes from public consultation are now used as final opinions at the risk evaluation phase. Unlike the former procedure on the verification of validity of Notification, the Commission can now consult MS and the EFSA, which will deliver a publicly available opinion in a period no longer than 30 days. These provisions do not affect the technical evaluation of the safety of a novel or traditional food, although they promote the inclusion of more players in a process that is expected to bring more clarity, efficiency, and coherence [80,81].

## 7. Perspectives for the EU Microalgae Sector

Microalgae have been recognized has an important player in the EU Green Deal and in particular for the farm-to-fork strategy; however, the novel foods framework initially delivered by the GFL is commonly seen as a commercialization barrier [82]. This idea is partially true; however, the lack of a tradition of microalgae-based products as food in the EU limits our knowledge on the real safety of these products [5]. As observed on the previous sections, microalgae-based products use in the food sector can be divided into two main groups: ones already consumed to a significant extent within the European territory before 15 May 1997 and ones completely new and demanding safety approval. Food supplements, contrarily, were regulated three years later and barely control the presence of biologically active substances, which leaves the decision to the interpretation of each MS. *Chlorella* and *Spirulina* are widely commercialized food supplements that are part of the safe list of novel foods from prior to 1997 regulation. However, these products are mostly sourced from outside Europe to a single market, entering under the FNOA status requiring a phytosanitary certificate, which is safety tracking of the origin of the product and not necessarily related to the quality of the content. Potential hazards present in the food supplements study case (Section 3.1.1.) have been identified as originating from production conditions. A wild-harvested North Sea seaweed study has detected cultivation, handling, processing and seaweed testing as key factors in food safety [83]. The geographical location of food products is considered not only an issue for microalgae-based products [84]. The use of isotope ratio mass spectrometry can be applied to microalgae-based products based on existing methods for commodities (e.g., cheese, wine, olive oil) [85]. The development of such an approach could better target the origin and therefore the quality, reducing the risk associated with imported products and sustain the quality of products originating from the EU. 

Prior to the Brexit situation, the UK, in the context of the BBSRC PHYCONET funded project, developed algal biotechnology guidelines for research and commercialization purposes (“Importing algae from outside the EU—clarifying UK legal requirements for the algal biotechnology industry (2018)”) [86]. This document bridges the gap between the recommendations of existing international agreements, such the International Plant Protection Convention (IPPC), and the absence of the term “algae” in the former directive that controlled the introduction of organisms harmful to plants or plant products and against their spread within the Community (Directive 2000/29/CE). The current law in force does not seem to put “algae” on the scope regarding the protective measures against pests of plants (Commission Implementing Regulation (EU) 2019/2072). At the EU level, academia and industry are partnering together to protect and promote a European algae industry. A Technical Committee, CEN TC/454 “Algae and algae products”, was created upon EC request to the European Committee for Standardization (CEN) to draft the European Standards to support the implementation of Art. 3, Directive 2009/28/EC, for algae and algae-based products. The development of such standards is expected to equilibrate the international competition of major producers and foster a high-quality seal for microalgae-based products, including for food purposes. At the same time, the European Algae Biomass Association (EABA) aims to promote a competitive yet integrative environment of any algae player in Europe [87]. 

The introduction of novel microalgae-based products on the market under the Authorization procedure has gained some attention due to its centralization process and applicant friendly approach, although the associated costs can burden the legalization of these novel foods products. In fact, the NFR 2015 implementation led to a considerable increase of the number of applications for the whole list of categories; however, few were related to microalgae-based products [88]. The GFL recent fitness check brings major advantages for EU consumer and the EU risk evaluators; however, it might bring some constraints for the EU microalgae industry in particular. The notification of studies implies a time-course planning that might affect this transition period if the acceptance of already performed studies do not have justification. The advisory meetings with the EFSA can benefit the industry, which is highly represented by SMEs. Despite the central role attributed to the EFSA, the decisions from third parties now have more impact. Confidentiality is a delicate aspect in an industry that is driven by technological innovation and intellectual property protection, yet the application of Transparency Regulation must protect human and animal health, as well as the environment, from risk. 

The joint application of national and private entities for the common Authorization of a product stands as an opportunity for a more cooperative industry and the potential consolidation of Europe as an important player in the global microalgae sector. The history of safe microalgae consumption outside Europe should be seen as an opportunity for the introduction of new species in the European market, drawing from the Asian and South American traditional microalgae diets. 

To date, there is not a clear map of the European microalgae production volume due to its small scale when compared to the impact of other continents [1,9]. Innovations at the end-user level are expected to drive the research of existing legal strains. Naturally occurring mutants, generated mutants via random mutagenesis, and site-directed mutagenesis can produce non-GMO organisms [21]. The generation of mutants from microalgae with QPS status can help to enhance product performance without compromising the safety of the producing organism. Mutants with low chlorophyll and high protein content of *Chlorella vulgaris* were obtained from chemically induced random mutagenesis [89]. The EFSA plays an important role as the central evaluation entity, and the methodologies for safety assessments should be adapted for the microalgal matrix through close cooperation between the industry and academia.

In order to promote the maturation of the European algae industry, several considerations should be taken into account at the moment of legislation:

- The existence of free entry point for imported microalgae-based products which can impact the health of the EU consumer;
- The small size of European algae industry;
- NFR 2015 associated costs;
- The diversity of microalgae-based products production processes;
- The lack of a tradition of evaluating the safety of microalgae-based products;
- Undefined adaptation period to the Transparency Regulation impacts on NFR 2015;
- Carbon neutrality technologies and ecosystem services that can play an important role in the European Green Deal.

## 8. Conclusions

Microalgae-based products can have an important role in the bioeconomy by providing various food ingredients through a low carbon emitting industry. The low number of microalgae-based products in the EU markets is an expression of a limited tradition. However, for some time, the premise of a highly nutritious food source has made microalgae available as food supplements, serving as a clear entry point to the market and leaving a record of consumption that still needs clarification among MS. The NFR 2015 update now includes food supplements as part of their scope, changing the safety assessment requirements. This measure promotes the safety of future novel food supplements; however, the importation of authorised microalgae food supplements is ongoing and attention should be paid thereto since the quality of these products is questionable. 

In such a complex legislative framework, EU microalgae will continue to struggle to get the expected importance it deserves. As the EFSA’s role becomes more central, the differences between MS harmonize, although costs associated to safety assessments restrict certain SMEs. In the end, the Transparency Regulation was designed to get a closer relationship with those smaller clusters, and at the same time provide disclosure to the EU consumer. 

This review has brought clarity about the EFSA’s concept of food safety and on the preparation of data to sustain those arguments. Opportunities were highlighted and were only possible through the elaboration of this document. We suggest that the capacitation of microalgae stakeholders at the legal level will strengthen the application of these microorganisms within the food sector. For example, industrial research can be more focused on EFSA requirements, while at the same time related non-microalgae entities will be empowered to raise their understanding of this new food source.

## Figures and Tables

**Table 1:** List of microalgae-based products on the EU market with a consumption history before 15 May 1997, according to the NFC (accessed on 22 February 2023).

Species	Organism Phylum
*Aphanizomenon flos-aquae* (AFA) var. *flos-aquae*	Cyanobacteria
*Arthrospira platensis*	Cyanobacteria
*Auxenochlorella protothecoides*	Chlorophyta
*Auxenochlorella pyrenoidosa*	Chlorophyta
*Chlorella luteoviridis*	Chlorophyta
*Chlorella pyrenoidosa*	Chlorophyta
*Chlorella sorokiniana*	Chlorophyta
*Chlorella vulgaris*	Chlorophyta
*Heterochlorella luteoviridis* (former *C. luteoviridis*)	Chlorophyta
*Jaagichlorella luteoviridis,*	Chlorophyta
*Parachlorella kessleri* (former *C. kessleri*)	Chlorophyta
*Spirulina* sp.	Cyanobacteria

**Table 2:** List of microalgae-based products authorized as novel foods with non-exhaustive product specifications [56].

NF	Organism Class	Relevant Specifications/Process
Microalgae oil of *Ulkenia* sp.	Labyrinthulomycetes	DHA > 32%
Astaxanthin-rich oleoresin from *Haematococcus pluvialis* algae	Chlorophyceae	Production can be in closed photobioreactor (PBR) with sunlight or open ponds with artificial illumination. Extraction can be done with supercritical CO_2_ or organic solvent (ethyl acetate).
Dried *Euglena gracilis*	Euglenaceae	*Euglena gracilis* is produced by fermentation, then filtered and thermally inactivated. Authorized on 23 December 2020 and confidentiality has been requested (Art. 26, Reg. 2283/2015) providing exclusivity to Kemin Foods L.C (2100 Maury Street, Des Moines, IA 50317, USA). until 23 December 2025.
*Odontella aurita* Microalgae	Bacillarrophyceae	Silicon: 3.3%
*Schizochytrium* sp. oil rich in DHA and EPA	Labyrinthulomycetes	DHA > 22.5% EPA > 10%
*Schizochytrium* sp. (ATCC PTA-9695) oil	Labyrinthulomycetes	DHA > 35%
*Schizochytrium* sp. (FCC-3204) oil	Labyrinthulomycetes	DHA > 32%
*Schizochytrium* sp. oil	Labyrinthulomycetes	DHA > 32%
*Schizochytrium* sp. (T18) oil	Labyrinthulomycetes	DHA > 35%
*Schizochytrium* sp. (WZU477) oil	Labyrinthulomycetes	DHA > 32
Dried *Tetraselmis chuii* microalgae	Chlorophyceae	Cultivated in marine seawater closed PBR. Label shall include the presence of negligible amounts of iodine.

**Table 3:** Novel foods related contact list of national competent authorities. EnhanceMicroAlgae partner countries were used as examples (adapted from [58]).

Country	National Competent Authority
Portugal	Direção Geral de Alimentação e Veterinária—DGAV (Directorate-General of Food and Veterinary—of Ministry of Agriculture) Campo Grande, nº 50 1700-093 LISBOA Portugal www.dgav.pt dsna@dgav.pt (accessed on 9 September 2023)
Spain	Agencia Española de Consumo, Seguridad Alimentaria y Nutrición (AECOSAN) Ministerio de Sanidad, Servicios Sociales e Igualdad c/ Alcala 56 E-28071 MADRID sgpsa@msssi.es https://www.aesan.gob.es (accessed on 9 September 2023)
Ireland	Food Safety Authority of Ireland The Exchange George’s Dock, I.F.S.C. Dublin 1 Ireland D01 P2V6 novelfood@fsai.ie www.fsai.ie (accessed on 9 September 2023)
France	DGCCRF—Bureau 4A Teledoc 223 59, boulevard Vincent Auriol F-75703 Paris Cedex 13 Tel.: 01 44 97 31 51 Bureau-4A@dgccrf.finances.gouv.fr https://www.entreprises.gouv.fr/fr (accessed on 9 September 2023)

**Table 4:** Resume of categories required for technical characterisation of EFSA’s evaluation of a novel food under the Authorization process, Art. 10, or traditional food under the Notification process, Art. 14, of NFR 2015. Caption x represents the category required in each of the novel foods application.

Category	Authorization	Notification
Resume (food source, basics of production, compositional features, purpose and intended use)	x	x
Identity of the novel food	x	x
Production process	x	x
Compositional data	x	x
Specifications	x	x
History of the use of NF and/or its source	x	
Data from experience of continued use		x
Proposed uses and use level and anticipated intake	x	
Proposed conditions of use for the EU market		x
Absorption, distribution, metabolism, and excretion (ADME)	x	
Nutritional information	x	
Toxicological information	x	
Other studies	x	x

## Data Availability

Not applicable.

## Abbreviations

ADME	Absorption, distribution, metabolism and excretion
AFA	*Aphanizomenon flos-aquae*
CEN TC	European Committee for Standardization Technical Committee
CARG	Compound Annual Growth Rate
EABA	European Algae Biomass Association
EMA	European Medicines Agency
EU	European Union
EC	European Commission
EFSA	European Food Safety Agency
EU	European Union
FBO	Food Business Operator
FNOA	food of non-animal origin
GCP	Good Clinical Practice
GLP	Good Laboratory Practice
GFL	General Food Law
GMO	Genetically Modified Organism
GMP	Good Manufacturing Practice
GRAS	Generally Recognized as Safe
ICH	International Council for Harmonisation of Technical Requirements for Pharmaceuticals for Human Use
IPCC	International Plant Protection Convention
ISO	International Organization for Standardization
MS	Member States
NDA	Panel on Nutrition, Novel Foods and Food Allergens
NFR	Novel Food Regulation
NFC	Novel Foods Catalogue
NFR 1997	Novel Food Regulation (EC) No. 258/1997
NFR 2015	Novel Food Regulation (EC) No. 2015/2283
OECD	Organization for Economic Co-operation and Development
PBR	Photobioreactor
PUFA	polyunsaturated fatty acids
QPS	Qualified Presumption of Safety
RASF	Rapid Alert System for Food and Feed
SCoPAFF	Standing Committee on Plants, Animals, Food and Feed
SME	Small and Medium Enterprises
Transparency Regulation	Regulation (EC) 2019/1381
